# Noninvasive Positive Pressure Ventilation against Reperfusion Pulmonary Edema following Percutaneous Transluminal Pulmonary Angioplasty

**DOI:** 10.1155/2011/204538

**Published:** 2012-01-05

**Authors:** Kiyoshi Moriyama, Sayuri Sugiyama, Koji Uzawa, Mariko Kotani, Toru Satoh, Tomoko Yorozu

**Affiliations:** ^1^Department of Anesthesiology, Kyorin University School of Medicine, 6-20-2 Shinkawa, Mitaka, Tokyo 181-8611, Japan; ^2^Department of Geriatric Medicine, Kyorin University School of Medicine, 6-20-2 Shinkawa, Mitaka, Tokyo 181-8611, Japan; ^3^Second Department of Internal Medicine, Kyorin University School of Medicine, 6-20-2 Shinkawa, Mitaka, Tokyo 181-8611, Japan

## Abstract

A 69-year-old man with chronic thromboembolic pulmonary hypertension (CTEPH) was on amblatory oxygen inhalation therapy (3 L/min) and scheduled for percutaneous transluminal pulmonary angioplasty (PTPA). The patient's New York Heart Association functional status was class III with recent worsening of dyspnea and apparent leg edema. Transthoracic echocardiography revealed right ventricular enlargement with mean pulmonary artery pressure of 42 mmHg. After PTPA, he was complicated with postoperative reperfusion pulmonary edema, and noninvasive positive pressure ventilation (NPPV) was applied immediately. Hypoxemia was successfully treated with 15 days of NPPV. Although mean pulmonary artery pressure was unchanged, his brain natriuretic peptide level decreased from preoperative 390.3 to postoperative 44.3 pg/dL. In addition, total pulmonary resistance decreased from preoperative 18 to postoperative 9.6 wood unit*·*m^2^. The patient was discharged on day 25 with SpO_2_ of 95% on 5 L/min of oxygen inhalation. Because pulmonary edema is a postsurgical life-threatening complication following PTPA, application of NPPV should be considered.

## 1. Introduction

Pulmonary hypertension is classified into 5 categories according to specific therapeutic interventions directed at the underlying causes [1]. Chronic thromboembolic pulmonary hypertension (CTEPH) is classified into WHO group 4, in which surgical procedures are recommended in selected patients [2]. We experienced a patient with CTEPH, who underwent percutaneous transluminal pulmonary angioplasty (PTPA). This patient was complicated with postoperative pulmonary edema, and noninvasive positive pressure ventilation (NPPV) was applied for the postoperative 15 days.

## 2. Case

A 69-year-old man with CTEPH was scheduled for PTPA; a new treatement strategy originally reported in 2001 [3] in USA and now developing in Japan [4]. On medical checkup at 52 years of age, he was pointed out to have cardiomegaly. At 54, he noticed exercisintolerance and dyspnea and was diagnosed to have pulmonary hypertension with cardiac catheterization and multiple stenoses and occlusions of his peripheral pulmonary arteries by pulmonary angiography. Pulmonary artery endarterectomy was not indicated because most of the lesions were not located at the proximal pulmonary arteries. He started to take warfarin, ticlopidine hydrochloride, and nifedipine. At 59, he had dyspnea at rest and was diagnosed to have protein S deficiency and CTEPH. His symptom developed gradually, and amblatory oxygen inhalation therapy (3 L/min) and oral intake of sildenafil citrate were started at 68 year, and PTPA was scheduled at 69.

On admission to our hospital, the patient's New York Heart Association functional status was class III with recent worsening of dyspnea and apparent leg edema. His SpO_2_ was 94% with 3 L/min of oxygen inhalation, cardiothoracic ratio on chest X-ray was 60%, and his brain natriuretic peptide (BNP) level was 390.3 pg/dL. Transthoracic echocardiography revealed right ventricular enlargement with mean pulmonary artery pressure of 42 mmHg. The diameter of the inferior vena cava (IVC) was 20/18 mm.

**Figure 1:**
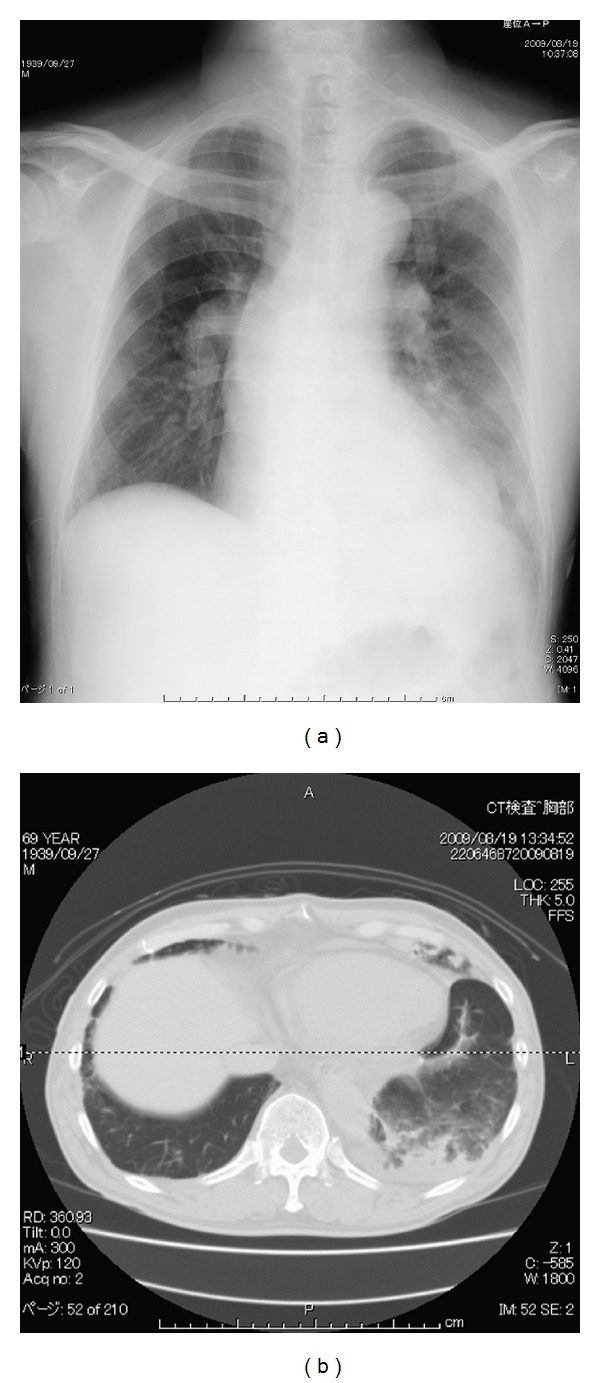
Chest X-ray and representative computed tomography image obtained on day 1. The patient had localized consolidation on his left lower lobe, suggesting postsurgical pulmonary edema in the dilated segment.

Initial PTPA was performed for his left pulmonary artery. Seven peripheral regions of his left pulmonary artery was dilated by the balloon. At the end of the procedure, his pulmonary artery pressure was 91/27 mmHg, and he was admitted to the intensive care unit. On admission to the intensive care unit (day 0), his SpO_2_ was 95% with 40% of oxygen inhalation with no complain of dyspnea. On day 1, his chest X-ray and computed tomography revealed localized consolidation on his left lower lobe (Figure 1). On day 2, consolidation on chest X-ray developed in accordance with his complication of dyspnea. Because his SpO_2_ decreased to 70% with 10 L/min of oxygen inhalation using reservoir mask, NPPV was applied.

The initial setting of NPPV was CPAP mode with 10 mmHg inhaling 100% of oxygen. Applying NPPV, SpO_2_ increased up to 90%. CPAP was gradually decreased and was 5 mmHg on day 5. NPPV was finally discontinued on day 16. Although mean pulmonary artery pressure was unchanged as assessed by postoperative cardiac catheterization, his brain natriuretic peptide level decreased from preoperative 390.3 to postoperative 44.3 pg/dL. In addition, total pulmonary resistance decreased from preoperative 18 to postoperative 9.6 wood unit·m^2^. The patient was discharged on day 25 with SpO_2_ of 95% on 5 L/min of oxygen inhalation and NYHA class II status.

## 3. Discussion

For patients suffering from CTEPH, pulmonary thromboendarterectomy (PTE) is increasingly successful [2]. Evidence-based clinical practice guidelines have recommended that patients with suspected CTEPH should be referred to centers experienced in the procedure for consideration of PTE [1]. However, not all patients with CTEPH have surgically accessible disease. In patients who are deemed nonsurgical or high-risk surgical candidates, there are 3 potential options: medical therapy, lung transplantation, and balloon pulmonary angioplasty (PTPA), a catheterization-based interventional management strategy [5].

Feinstein et al. reported 18 patients without surgical potential who underwent PTPA [6]. In their report, the average New York Heart Association class improved from 3.3 to 1.8 after an average of 36 months of followup. However, 11 patients suffered from postsurgical pulmonary edema defined as radiographic opacity in the dilated segment and worsening hypoxemia, 4 at the time of catheterization and 7 during the subsequent 48 hours. All patients with reperfusion pulmonary edema were managed with diuretics and oxygen. Three patients required mechanical ventilation and 1 patient died 1 week after PTPA.

In the case presented, the patient started to suffer from postsurgical pulmonary edema in the dilated segmenton on day 1. To avoid mechanical ventilation for worsening hypoxemia, NPPV was immediately applied, and oxygenation was improved. After 16 days of NPPV treatment, his right ventricular dysfunction improved as evidenced by decreased BNP concentration [7].

In summary, we experienced a patient with postsurgical, reperfusion pulmonary edema following PTPA. The patient developed severe hypoxemia and was successfully treated with 15 days of NPPV. Because pulmonary edema is a postsurgical life-threatening complication following PTPA, application of NPPV should be immediately considered.
